# Patient Satisfaction in Primary and Specialised Ambulatory Healthcare: A Web-Based Cross-Sectional Study in the Polish Population

**DOI:** 10.3390/healthcare13101147

**Published:** 2025-05-14

**Authors:** Agnieszka Pochrzęst-Motyczyńska, Janusz Ostrowski, Dorota Sys, Jarosław Pinkas, Urszula Religioni

**Affiliations:** 1 School of Public Health, Centre of Postgraduate Medical Education, Kleczewska 61/63, 01-826 Warsaw, Poland; 2 Department of Biochemistry and Molecular Biology, Centre of Postgraduate Medical Education, Marymoncka 99/103, 01-813 Warsaw, Poland; dorota.sys@cmkp.edu.pl

**Keywords:** patient satisfaction, healthcare quality, communication

## Abstract

**Background:** Patient satisfaction is one of the key patient-reported indicators of healthcare quality. In the study, we assess satisfaction with visits to primary healthcare (POZ) and specialised ambulatory healthcare (AOS). **Methods:** This web-based cross-sectional study was conducted in a representative sample of 725 patients from the Polish population in June 2023. The study employed the Patient Expectations Scale, comprising 18 statements addressing various aspects of a medical visit. **Results:** The average satisfaction rating for the medical visit was 7.41 (±2.34) out of 10, with a median of 8. Strong correlations were found between overall visit satisfaction and specific aspects of the doctor–patient interaction. The highest correlations were observed for expressions of empathy and support, such as “showed concern” (r = 0.73) and “encouraged me” (r = 0.68), as well as for clear communication about treatment (“presented a probable course of treatment” (r = 0.62) and disease consequences (r = 0.55). Presenting test results (r = 0.51) and treatment recommendations (r = 0.63) were also significantly associated with overall satisfaction (all *p* < 0.001). **Conclusions:** This study shows that patients reported higher satisfaction with specialised ambulatory care (AOS) than with family medicine (PR), mainly due to better communication, encouragement and concern from AOS doctors. Improving healthcare quality in Poland requires not only financial and organisational efforts but also a focus on patient expectations, supported by regular use of satisfaction measurement tools.

## 1. Background

According to the World Health Organisation (WHO), the quality of medical care and patient safety is the most important priority in healthcare. WHO has developed the Global Patient Safety Action Plan for 2021–2030—Pathway [1] to eliminating avoidable harm to health. Medical facilities should strive for quality and increase the standard of services provided. One of the changes that need to be introduced is the training of skills such as listening and communication with the patient and the team. According to WHO experts, such skills are necessary to provide safe and high-quality care, achieve the best diagnosis and treatment results and increase patient satisfaction scores.

The definition of quality in healthcare has evolved over the years. In the 1980s, it was emphasised that the basis of high-quality healthcare was doing the right thing at the right time, in the right way, for the right person, and achieving the best possible outcomes [2]. When talking about quality in healthcare, we cannot focus only on technical factors, but also organisational (patient service), psychological (relationship between the patient and doctor), social and economic (possibility of providing additional services) factors [3]. All these elements determine the high quality of health services. The quality features of medical services also include reliability, sensitivity, appearance, availability, competence, courtesy, transparency, responsibility, communication and safety [4].

Quality measurement is particularly important for a primary healthcare program, especially in developing countries, because without quality assessment, resources will not be used effectively [5]. Recent research indicates that healthcare quality management is also influenced by several key elements of communication (clear, logical reporting narratives, open communication, effective questioning and challenge from members) and leadership that is focused on excellence in care health and quality improvement [6].

According to researchers, the value of care ultimately must be assessed by the person receiving the care and cannot be defined solely by clinicians [7]. They indicate that in addition to clinically reported outcome measures, experience in the care process also counts. They emphasise that patient-reported experience measures (PREMs) differ from patient-reported outcome measures (PROMs) because PREMs refer to the conditions in which healthcare is delivered (structure) and the interactions between patients and providers (process), while PROMs refer to the impact of healthcare on the health of patients and populations (outcomes). According to researchers, a common feature of both approaches is the need to understand the needs and desires of people receiving care [7]. The rationale is that the better patients are understood, the better their experience of the structure, processes and outcomes of the healthcare system will be. As a result, it is expected to improve loyalty, adherence, satisfaction and, ultimately, clinical outcomes.

Researchers from Boston point out that modern healthcare is “patient-centric” and “consumer engagement”. They indicate that well-organised team care also influences the level of patient satisfaction [8].

In Poland, there are no representative studies on the satisfaction of patients using primary and specialised healthcare, taking into account various socio-demographic factors. Polish researchers analysed whether place of residence affects patient satisfaction with hospital healthcare [9]. Mean scores on the 28 satisfaction items on a scale of 1–5 were similar between rural and urban samples and generally showed a trend toward positive experiences. The analysis identified significant associations between place of residence and patient satisfaction with respect to three components of hospital care: (1) hospital conditions and staff care, (2) professional skills of physicians and (3) hospitalisation outcomes (e.g., improvement in health condition and emotional state). After taking into account socio-demographic variables, the relationship remained significant only in terms of satisfaction with the results of hospitalisation.

Researchers from Wrocław investigated the association between satisfaction with doctor–patient communication and adherence to treatment and self-care among patients with chronic conditions. Their study focused on 250 individuals diagnosed with hypertension [10]. The findings revealed that higher satisfaction with communication significantly correlated with better adherence to medical recommendations and more proactive self-care behaviours. In other words, patients who perceived communication with their physician as effective and supportive were more likely to follow treatment plans and engage in health-promoting activities.

Research conducted among oncology patients also showed that good communication between medical staff and the patient is necessary to provide appropriate support but is also a key determinant of patient satisfaction [11]. To improve communication, it is necessary to understand the factors that determine satisfaction or lack thereof in doctor–patient communication. The authors emphasise that the communication competences not only of medical staff, but also of patients, are important in this relationship.

In Poland, people started talking about quality in the 1990s, but the topic is still relevant and requires introducing systemic changes [12]. Competition between facilities means that the quality of services has become a priority and one of the primary goals of the facilities’ operation. As foreign studies show, patient satisfaction has become one of the key patient-reported indicators of healthcare quality [7], but there are no representative studies in Poland. We are at a key moment in the development of the Polish healthcare system. A pilot of coordinated care in primary healthcare is underway, and in 2023, to improve the quality of medical services, the Act on Quality in Healthcare and Patient Safety came into force [13]. It introduces new requirements for hospital managers. Quality is to be defined and measured by indicators relating to the following areas: clinical, consumer and management. Facilities must implement solutions that identify the risk of adverse events and methods of managing this risk, as well as identify priority areas for improving the quality and safety of the services provided. What is new is that units will also have to conduct research on patients’ opinions and experiences based on a survey, the template of which will be published by the Minister of Health in the Public Information Bulletin.

Taking into account the above, the aim of our research was to verify the level of patient satisfaction after a medical visit in primary and specialised healthcare in Poland based on questionnaires. We also analysed the correlations between sociodemographic indicators and the level of satisfaction with a medical visit in primary care and emergency care.

## 2. Methods

### 2.1. Study Design

This web-based cross-sectional study was conducted in a representative sample of 725 patients from the Polish population in June 2023. Data were collected using the CAWI (Computer-Assisted Web Interview) method. This article has been written in accordance with the STROBE (Strengthening the Reporting of Observational Studies in Epidemiology) guidelines [14] and the CHEERIES (Checklist for Reporting Results of Internet E-Surveys) framework [15].

### 2.2. Setting

The study was conducted online by Nationwide Research Panel Ariadna (See further datalis: https://panelariadna.pl/regulamin.pdf?v=10052024 accessed on 10 May 2025), targeting the Polish population. The data collection period was June 2023.

### 2.3. Participants

The study included a representative sample of the Polish population. The inclusion criteria were age over 18 years and a visit to a medical doctor within the last three months. Participants were selected based on a stratified sampling model from a pool of over 100,000 registered and verified individuals on the Ariadna platform. If a chosen respondent declined to participate, another respondent meeting the inclusion criteria was randomly selected to maintain population representativeness.

### 2.4. Variables and Data Measurement

The research tool was a questionnaire—the Patient Expectations Scale, developed for the Promoting Active Ageing PRACTA project. (PRACTA is a Polish–Norwegian research project whose aim was to activate older people in medical practice. The study consisted of a baseline questionnaire, implementation of the intervention and a follow-up questionnaire administered 1 month after the intervention.) The questionnaire consisted of 18 statements relating to various elements of a visit to a doctor. The authors of the article obtained consent to use this questionnaire. The original authors of the scale assessed the validity and reliability of the tool in the Polish population [16]. Each statement had a 7-point response scale. The study participant (patient) marked the square next to the number that best expressed the degree to which a given element of the visit most closely reflected the situation during a medical visit. The higher the indicated score, the more the patient’s expectations were met during the visit. Additionally, respondents completed a form in which they indicated the main sociodemographic characteristics, such as age, gender, education, marital status and place of residence.

### 2.5. Study Size

The study included 1062 individuals, of which 725 participants responded affirmatively to having visited a medical doctor in the past three months. These respondents met the inclusion criterion for the study and subsequently completed the PRACTA questionnaire.

### 2.6. Statistical Analysis

Data were collected using questionnaires and then analysed using the Statistica statistical package v13. Descriptive analysis was employed to present the demographic characteristics of the study participants. Means and standard deviations were calculated for individual statements in the PRACTA questionnaire and the overall satisfaction rating. Correlation analysis was conducted to assess the relationships between different variables included in the questionnaire. Analysis of variance (ANOVA) was performed to compare the mean scores questionnaire and overall satisfaction ratings across various demographic groups. Additionally, *t*-tests were used to compare the mean scores of individual questionnaire items between visits in primary care and specialist outpatient care. A significance level of *p* < 0.05 was adopted as the threshold for statistical significance.

### 2.7. Ethics Consideration

The participants were informed that they were participating in a study, and completing the questionnaire implied their consent to participate. This study received consent from the Bioethics Committee at the Medical Centre of Postgraduate Education in Warsaw (No. 197/2023 of 24 May 2023).

## 3. Results

### 3.1. Characteristics of the Study Group

The study group included 725 people, 56% of whom were women and 44% men. The largest percentage of participants was over 55 years old (36.69%), while the smallest age group was of people aged 18–24 (11.03%). In terms of marital status, married people dominated (67.59%), and widowers were the least common (4.28%).

Most participants had higher education (40.14%) or secondary education with high school leaving exams (36.00%), while only 2.76% had primary education. With regard to place of residence, the largest part of the respondents lived in rural areas (35.45%), and the smallest in small towns (11.86%). As for the housing situation, most people lived with their spouses (64.41%), and the least with their grandchildren (1.93%). The majority of respondents (62.76%) were working people. In the context of the financial situation, most people rated it as average (52.28%), and the least as very good (5.24%).

As for the current health condition, the majority of respondents rated it as average (42.07%) or good (38.48%). Regarding the number of diseases, the largest group had no diseases (24.55%), and the smallest group had three or more diseases (5.38%). The group was divided almost in half according to the type of medical visits: visits within specialised healthcare constituted 54.90%, while visits to primary care/family medicine constituted 45.10% of all visits. Detailed data on the characteristics of the group are provided in Table 1.

### 3.2. Patients’ Expectations

**Figure 1:**
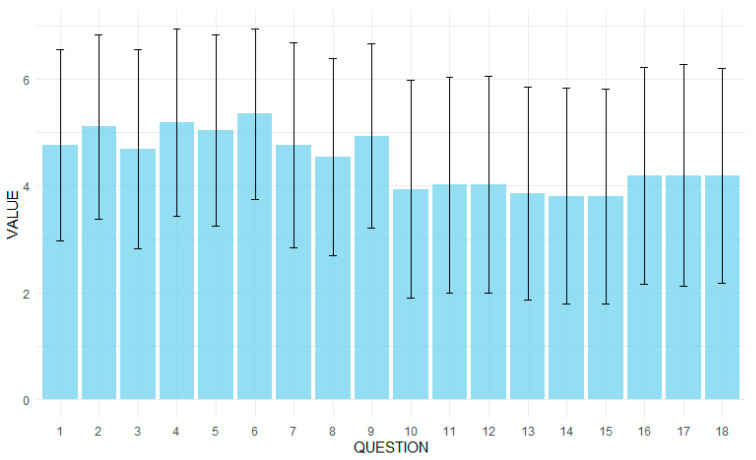
Results of the PRACTA questionnaire.

Figure 1 shows the results of the PRACTA questionnaire and the overall assessment of satisfaction with the doctor’s visit among 725 respondents. The average rating for determining the cause of the symptoms was 4.77 (±1.79), with half of the respondents rating this issue as 5 or higher. The presentation of the probable further course of treatment had an average score of 5.11 (±1.73), and the presentation of test results—5.19 (±1.76), which indicates relatively high satisfaction in these aspects. Recommendations related to the treatment were rated the highest, with an average of 5.35 (±1.60). For medication advice and well-being discussions, mean ratings were 5.04 (±1.79) and 4.77 (±1.92), respectively. Advice on spending time actively and maintaining life satisfaction was rated the lowest, with an average score of 3.81 (±2.02) for both categories. The sum of the PRACTA questionnaire points had an average value of 80.40 (±19.00), with a maximum possible value of 126. The overall satisfaction score with the medical visit was on average 7.41 (±2.34) out of 10 possible points, with a median of 8.

**Figure 2:**
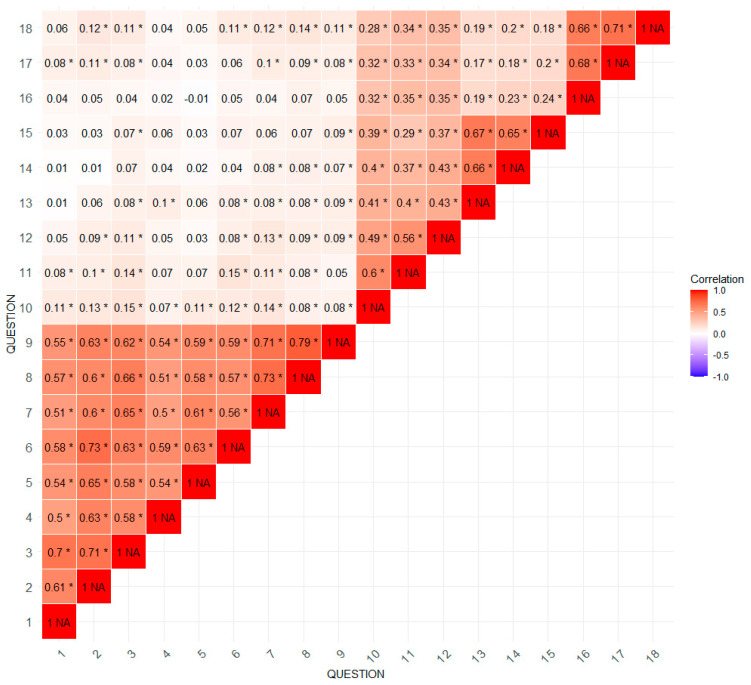
Correlation matrix of statements from the PRACTA form. NOTE: The numbers in the cells represent the values of the correlation coefficients between the corresponding survey statements (1–18). Cell colours indicate the strength and direction of the correlation: from strong positive correlations (dark red) through no correlation (white) to strong negative correlations (dark blue). Asterisks (*) next to correlation values indicate statistical significance (*p* < 0.05).

The correlation analysis between various aspects of the medical visit presented in Figure 2 reveals significant associations between the assessed variables. These correlations are typically moderate to high, suggesting that respondents’ responses to the various statements are related. Statements about presenting test results and the likely course of treatment have high correlations with other statements about information provided by the doctor, reflecting the significant interdependencies between these aspects of visits—for example, the correlation between statements 1 and 6 is 0.73. Statements about the doctor’s concern and empathy, such as “he showed care for me” and “he showed me respect”, also show high correlations; e.g., the correlation between 15 and 18 is 0.71. The table shows a concentration of high correlations in two areas: the upper triangle on the right and the lower triangle on the left. This suggests that statements in these areas are strongly interconnected internally. Statements about specific doctor activities (e.g., tests, treatment) and statements about emotional support are highly correlated within their groups but do not correlate with each other.

The numbers represent subsequent statements. During the last visit, the doctor 1—found the cause of my symptoms, 2—presented me with a probable further course of treatment, 3—discussed the possible consequences of the disease, 4—presented the results of the tests performed, 5—gave me advice on the medications I am taking, 6—presented recommendations related to the treatment, 7—talked to me about how I felt and how I was coping, 8—gave me encouragement, 9—showed me concern, 10—talked to me about what was harmful to my health, 11—advised me what I can do to improve my functioning in everyday life, 12—encouraged me to make favourable changes, 13—suggested how to maintain contacts with other people, 14—talked to me about how I can spend my time actively, 15—suggested how to maintain life satisfaction, 16—was friendly towards me, 17—treated me seriously, 18—showed me respect.

Analysis of the correlation between individual questionnaire statements, the total PRCTA score and the patient’s overall assessment of the visit reveals significant associations. Statements regarding specific aspects of the doctor’s visit, such as “he presented me with the probable further course of treatment” (r = 0.62, *p* < 0.001) and “discussed the possible consequences of the disease” (r = 0.55, *p* < 0.001), show high correlations with the overall assessment of the visit.

Statements related to the doctor’s concern and empathy, such as “he encouraged me” (r = 0.68, *p* < 0.001) and “showed concern for me” (r = 0.73, *p* < 0.0001), also have high correlations. Moreover, statements regarding the presentation of test results (r = 0.51, *p* < 0.001) and the presentation of treatment recommendations (r = 0.63, *p* < 0.001) are also significantly correlated with the overall assessment of the visit. However, statements related to more detailed aspects, such as “he talked to me about what is harmful to my health” (r = 0.11, *p* = 0.002) and “encouraged me to make favourable changes” (r = 0.08, *p* = 0.025), have low correlations. Statements about activity and life satisfaction, such as “he talked to me about how I can spend my time actively” (r = 0.05, *p* = 0.165) and “suggested how to maintain life satisfaction” (r = 0.04, *p* = 0.311), show very low correlations and are not statistically significant. The sum of the PRACTA questionnaire scores has a moderate correlation with the patient’s overall assessment of the visit (r = 0.59, *p* < 0.001).

The next step was to analyse the results of the PRACTA form and the overall assessment of satisfaction with the doctor’s visit depending on demographic data. The average results of the PRACTA questionnaire and the overall satisfaction rating were analysed in the context of various demographic categories, such as gender, age, marital status, education, place of residence, housing situation, employment, financial situation, current health status, number of diseases and type of medical visit.

With regard to gender, both women and men obtained very similar mean PRACTA scores (80.43 ± 19.20 for women and 80.46 ± 18.85 for men) and similar overall satisfaction ratings (7.37 ± 2.48 for women and 7.46 ± 2.16 for men), which indicates no significant differences in these categories (*p* = 0.98 and *p* = 0.62, respectively).

Analysis of the results in the context of age showed that the lowest results were achieved by people aged 45–54 (77.82 ± 18.75), while the highest results were achieved by people over 55 years of age (82.06 ± 18.83). Also, in the case of satisfaction assessment, the highest ratings were obtained by the 55+ age group (7.64 ± 2.41), although the differences were not statistically significant (*p* = 0.25 and *p* = 0.29).

Marital status appeared to have some impact on the results: separated or divorced people had the highest mean PRACTA scores (84.89 ± 20.47) and the highest satisfaction ratings (7.94 ± 2.38). Single people had the lowest results (77.51 ± 20.20). Despite these observations, the differences were not statistically significant (*p* = 0.08 and *p* = 0.13).

In terms of education, the highest average PRACTA scores were achieved by people with primary education (81.45 ± 17.86) and people with secondary education without a high school diploma (81.91 ± 19.13). The overall satisfaction rating was the highest in the group of people with primary education (7.90 ± 2.17), but the differences were also not statistically significant (*p* = 0.73 and *p* = 0.64).

The place of residence did not have a significant impact on the results of the PRACTA questionnaire or on the overall satisfaction rating (*p* = 0.76 and *p* = 0.57), as did the housing situation, employment and number of diseases. However, the financial situation had some impact on the results, where people assessing their situation as “rather bad” had lower PRACTA scores (77.06 ± 16.87) and overall satisfaction ratings (6.64 ± 2.66), which was statistically significant only in the case of overall satisfaction ratings (*p* = 0.01).

Type of medical visit showed significant differences, with secondary care visits having higher mean PRACTA scores (82.85 ± 18.71) and higher overall satisfaction scores (7.59 ± 2.36) compared to primary care/family medicine visits (*p* < 0.001 and *p* = 0.03) (Table 2).

After obtaining a statistically significant difference in the total points of the PRACTA questionnaire between visits to primary care and specialised ambulatory healthcare, it was decided to analyse the results obtained from individual statements of the questionnaire, in order to identify in detail the areas in which patients’ perception of visits to primary care and specialised ambulatory healthcare differ. Patients in AOS more often received detailed information about the further course of treatment (5.38 ± 1.66 vs. 4.79 ± 1.76, *p* < 0.001) and possible consequences of the disease (4.87 ± 1.85 vs. 4.46 ± 1.86, *p* = 0.002). Test results were presented in more detail in the AOS (5.51 ± 1.62 vs. 4.80 ± 1.84, *p* < 0.001), as were medication advice (5.18 ± 1.79 vs. 4.87 ± 1.79, *p* = 0.008) and treatment recommendations (5.59 ± 1.49 vs. 5.06 ± 1.68, *p* < 0.001).

Doctors in AOS talked to patients more often about their health condition and coping with the disease (4.97 ± 1.87 vs. 4.52 ± 1.95, *p* = 0.001), gave encouragement (4.70 ± 1.87 vs. 4.34 ± 1.81, *p* = 0.004) and showed concern (5.15 ± 1.69 vs. 4.69 ± 1.76, *p* < 0.001). In AOS, topics related to health hazards were discussed more often (4.11 ± 2.03 vs. 3.75 ± 2.04, *p* = 0.018), and patients were treated more seriously (4.36 ± 2.06 vs. 4.00 ± 2.09, *p* = 0.022). In all significantly different areas, higher scores were obtained by AOS, which confirms patients’ better perception of the quality of visits to AOS than to primary care (Table 3).

## 4. Discussion

This study analysed patient satisfaction and expectations related to recent medical visits among 725 adult respondents in Poland. The largest age group was individuals aged 55 and above, and more than half of the participants were women. Respondents most often rated their financial and health situation as average. Primary and specialist care visits were nearly evenly represented.

Overall, patients reported relatively high satisfaction with the visits, with an average rating of 7.41 out of 10. Recommendations related to treatment and test result explanations received the highest ratings, while lifestyle advice received the lowest. Notably, visits in specialist ambulatory care were rated significantly higher than those in primary care in almost all aspects of the visit, including communication, information provision and emotional support. Satisfaction levels were only moderately influenced by sociodemographic factors, with financial status and type of care being the most significant.

Research on patient satisfaction and theories on this subject began to emerge in the 1980s. They first began in the private sector when patients began to be viewed as consumers of healthcare. Patient satisfaction surveys can therefore be perceived as an effect of marketisation and the recognition of the need for dialogue between the service provider and the service recipient, i.e., the patient [17]. Patient satisfaction is a complex concept and depends on the subjective feeling of the patient. A. Donabedian includes them as healthcare services and emphasises that although some results are easy to measure, e.g., death, there are also those that are more difficult to verify, e.g., patient attitudes and satisfaction [18]. Satisfaction is the difference between what the patient receives and his subjective expectations [19]. If the difference is positive, the patient is happy and satisfied, but if it is negative, the patient feels dissatisfied with the service received.

“Patient satisfaction” is defined as the overall evaluation of the healthcare experience [20]. Another definition emphasises that patient satisfaction is a cumulative construct that includes technical, functional, infrastructural, interactional and atmospheric aspects [21]. Because each patient independently and voluntarily chooses the criteria that are important to him during the evaluation, the same service may be assessed differently by patients. People—having different hierarchies of values and needs—take into account other aspects of the medical service during the assessment.

Research on the relationship between quality attributes of outpatient medical services and patient satisfaction was conducted among Korean patients [22]. It turned out that four dimensions of service quality explain 50 percent of variance in the level of satisfaction. Staff care was a particularly important determinant of satisfaction. The other two dimensions are the course of the treatment process and medical care. Material elements turned out to be the least important in assessing patient satisfaction. These studies—like ours—showed that the patient–staff relationship is crucial in assessing patient satisfaction.

A study conducted in Great Britain showed that trust has the greatest contribution to creating patient satisfaction but also strongly influences other effects of doctors’ actions [23].

Demographic and social variables may also influence the level of patient satisfaction, but the results are not clear. Gender does not play a significant role in building patient satisfaction. However, the level of satisfaction with medical care increases with age [24]. This is also confirmed by the results of our research, which show that the lowest results were achieved by people aged 45–54, while the highest results were achieved by people over 55. Also, in the case of satisfaction assessment, the highest ratings were obtained by the 55+ age group, although the differences were not statistically significant.

A survey of 126 people who randomly visited the Hellenic Air Force Medical Branch between January and February 2019 found that healthcare users reported high levels of physician empathy and overall satisfaction but low levels of hospital satisfaction [25]. The more positively patients rated their doctor’s empathy, the more satisfied they were with other factors.

Other studies have shown that, in addition to doctors’ medical knowledge, patients valued their ability to listen [26], ease of communication and friendliness [27]. The key to achieving patient satisfaction is patient-centred communication [28], in which the doctor tries to understand the patient’s point of view on the disease and shows empathy. Such communication requires acknowledging the patient’s feelings, concerns and experiences regarding the effects of the disease. A doctor’s empathy can be expressed by naming the feeling, conveying understanding, respect and support and examining the experiences and emotions related to the patient’s illness.

In our study—in all significantly different areas—AOS obtained higher results than POZ, which confirms the patients’ better perception of the quality of visits to AOS than to POZ. This happened because outpatient specialist care patients were more likely to receive detailed information about the further course of treatment and the possible consequences of the disease. Test results and medication advice were also presented in more detail in the AOS. Doctors at AOS also talked to patients more often about their health condition and coping with the disease, encouraged them and showed concern. During a visit to a specialist clinic, topics related to health hazards were also discussed more often, but in the patients’ opinion, they were treated more seriously. As you can see, showing empathy and the doctor’s involvement in the relationship and appropriately providing information to the patient were crucial when it came to the level of patient satisfaction.

Another important factor influencing a patient’s satisfaction with a healthcare service is the waiting time for the service [29]. Researchers point out that the total waiting time for a doctor was the most important predictor of patient satisfaction. They indicate that informing patients how long they would wait were also significant predictors of patient satisfaction. The results of subsequent studies have shown that waiting times, even if they cannot be reduced, can be managed more effectively to improve patient satisfaction [30].

In our study, we focused on the communication competences of doctors, but a new challenge for staff, not only in the Polish healthcare system, is cultural competence. It is estimated that the total number of immigrants in Poland is approximately 3.5–4 million, of which 60–75 percent are Ukrainians [31]. Immigrants coming to Poland also come from Belarus, Georgia, India and Moldova, and since 2008, an important trend has been an increase in the share of refugees/immigrants coming to Europe from African and Middle Eastern countries. Poland is a border country of the European Union on the route of migrants from Asia and Africa transferred to the EU from Belarus as part of the hybrid war. As research from the United States shows, in order to improve the satisfaction of patients from minority groups, it is necessary to introduce training for healthcare workers in the field of cultural competences [32]. Studies have shown that such training increases the satisfaction of patients from minority groups.

In the survey, the results of which are presented in this article, the Patient Expectation Scale from the PRACTA project was used. The survey developed for the Promoting Active Ageing (PRACTA) study consisted of a baseline questionnaire, implementation of the intervention and a follow-up questionnaire that was administered 1 month after the intervention. A total of 151 primary care facilities and 503 general practitioners agreed to take part in the baseline assessment [33]. Doctors were divided into three groups: access to an e-learning platform with knowledge about the needs of older people and ways of activating them, a PDF article with the same content and a placebo group without such support. The study showed that both e-learning and the pdf article were effective, but in different areas and under different conditions. A key benefit of the pdf article intervention was to prompt physicians to reflect on limitations in their communication skills, while e-learning was more effective in changing perceptions of the proactive attitude of older patients, especially among GPs working in private settings and having a larger number of assigned patients.

Another study conducted as part of the PRACTA project analysed whether age had an impact on primary care patients’ expectations regarding medical visits [34]. Differences in patients’ expectations before medical visits depending on age were observed in the following factors: explanation of the disease, explanation of treatment, quality of life, relationships and emotional support. Such differences were not observed in the case of health promotion. Differences between pre- and post-visit measurements were statistically significant in all age groups. In all groups, the number of patients who received less from their doctors than they expected outweighed the number of patients who received what they expected or more.

### Study Limitations

This study has several limitations. Data were collected through an online survey, which may have excluded individuals with limited internet access or digital skills, introducing potential selection bias. Additionally, only respondents who had visited a doctor in the last three months were included, which may limit the generalisability of the findings to individuals with less frequent healthcare use, particularly older adults. The age structure of the sample may also underrepresent the oldest age groups with specific care needs. Finally, the results reflect the context of the Polish healthcare system and may not be directly transferable to countries with different healthcare models or patient expectations.

## 5. Conclusions

Research confirms that the patient’s overall satisfaction with the visit is strongly correlated with the experience of care and empathy from the doctor during the visit. Patients’ higher satisfaction with visits in AOS resulted from the fact that they more often received detailed information about the further course of treatment and possible consequences of the disease. The higher level of satisfaction was also influenced by the fact that in specialised healthcare, patients had tests presented in more detail and received more information about medications and treatment recommendations. Doctors at AOS were also more likely to talk to patients about their health and coping with the disease.

When improving the quality of health services provided in Poland, we must take into account not only the financial and organisational capabilities of the healthcare system but, above all, patients’ expectations. For this purpose, it is necessary to systematically introduce scales measuring patient satisfaction to obtain information on how the patient evaluates various elements related to the provision of services.

According to the Act on Quality in Healthcare and Patient Safety, medical facilities in Poland will have to conduct anonymised research on patients’ opinions and experiences. To improve the quality of medical services in Poland, it will be crucial how these surveys are constructed, because—as research shows—the level of satisfaction with health services is influenced by many factors, such as the patient’s age, level of education, waiting time for an appointment, marital status and the type of service provided. Conducting surveys is just the beginning of patient experience management. It is also necessary to systematically introduce a “feedback loop” [33] so that the comments submitted by patients are analysed and taken into account in changes planned in the Polish healthcare system.

Moreover, patient feedback should not remain at the level of aggregated statistics. It should be actively used in daily quality improvement processes at the facility level. This means involving medical staff in the interpretation of results, identifying actionable areas for change and co-creating solutions with both staff and patients. Such an approach can strengthen organisational learning, improve communication within teams and promote a culture of continuous improvement centred around the patient. Furthermore, satisfaction data should be integrated with other quality indicators—including clinical outcomes and safety metrics—to provide a holistic view of performance and guide strategic decision-making.

## Figures and Tables

**Table 1:** Characteristics of the study group.

Category	Subcategory	N	%
**Sex**	Female	406	56.00
	Male	319	44.00
**Age**	18–24 years	80	11.03
	25–34 years	142	19.59
	35–44 years	106	14.62
	45–54 years	131	18.07
	55+ years	266	36.69
**Marital status**	Miss/bachelor	142	19.59
	Separated/divorced	62	8.55
	Widow/widower	31	4.28
	Married	490	67.59
**Education**	Primary	20	2.76
	Vocational	60	8.28
	Secondary school, no final exams	93	12.83
	Secondary school, with exam	261	36.00
	Higher	291	40.14
**Domicile**	Very big city (500+ thousand)	100	13.79
	Big city (100–500 thousand)	130	17.93
	Average city (20–99 thousand)	152	20.97
	Small city (under 20 thousand)	86	11.86
	Village	257	35.45
Lives with: *	Alone	87	12.00
	Spouse	467	64.41
	Children	251	34.62
	Grandchildren	14	1.93
	Other members of family	170	23.45
	Other persons	15	2.07
**Working person**		455	62.76
**Financial situation**	Good	38	5.24
	Rather good	173	23.86
	Average	379	52.28
	Rather bad	94	12.97
	Bad	41	5.66
**Current health status**	Very good	21	2.90
	Good	279	38.48
	Average	305	42.07
	Bad	106	14.62
	Very bad	14	1.93
**Number of diseases**	None	178	24.55
	1 disease	237	32.69
	2 diseases	177	24.41
	3 diseases	94	12.97
	More	39	5.38
**Type of visit**	POZ/family medicine	327	45.10
	AOS/specialised healthcare	398	54.90

* Multiple choice question, does not add up to 100%.

**Table 2:** Form score depending on demographic data.

Category	Subcategory	Sum (M ± SD)	Statistic (*t*-Test or ANOVA)	*p*-Value (sum)	Overall Rating (M ± SD)	Statistic (*t*-Test or ANOVA	*p*-Value (Overall Rating)
**Sex**	Female	80.43 ± 19.20	−0.02	0.98	7.37 ± 2.48	−0.49	0.62
	Male	80.46 ± 18.85			7.46 ± 2.16		
**Age**	18–24 years	78.75 ± 20.13	1.36	0.25	7.11 ± 2.37	1.24	0.29
	25–34 years	80.04 ± 20.44			7.25 ± 2.19		
	35–44 years	81.46 ± 16.84			7.47 ± 2.18		
	45–54 years	77.82 ± 18.75			7.27 ± 2.44		
	55+ years	82.06 ± 18.83			7.64 ± 2.41		
**Marital status**	Miss/bachelor	77.51 ± 20.20	2.30	0.08	7.11 ± 2.37	1.87	0.13
	Separated/divorced	84.89 ± 20.47			7.94 ± 2.38		
	Widow/widower	81.23 ± 18.42			7.58 ± 2.66		
	Married	80.68 ± 18.46			7.42 ± 2.30		
**Education**	Primary	81.45 ± 17.86	0.52	0.73	7.90 ± 2.17	0.64	0.64
	Vocational	79.77 ± 21.76			7.43 ± 2.26		
	Secondary school, no final exams	81.91 ± 19.13			7.46 ± 2.11		
	Secondary school, with exam	79.26 ± 19.21			7.25 ± 2.49		
	Higher	81.11 ± 18.38			7.50 ± 2.30		
**Domicile**	Very big city (500+ thousand)	79.52 ± 18.31	0.46	0.76	7.28 ± 2.47	0.73	0.57
	Big city (100–500 thousand)	80.80 ± 20.36			7.18 ± 2.46		
	Average city (20–99 thousand)	78.96 ± 17.78			7.44 ± 2.15		
	Small city (under 20 thousand)	81.65 ± 20.00			7.67 ± 2.27		
	Village	81.10 ± 19.09			7.48 ± 2.37		
**Lives alone**	No	80.74 ± 18.71	1.03	0.26	7.46 ± 2.29	1.41	0.16
	Yes	78.28 ± 21.24			7.08 ± 2.67		
**Lives with spouse**	No	79.21 ± 20.94	−1.24	0.19	7.31 ± 2.49	−0.91	0.36
	Yes	81.13 ± 17.89			7.47 ± 2.25		
**Lives with children**	No	80.39 ± 19.79	−0.12	0.91	7.34 ± 2.41	−1.18	0.24
	Yes	80.56 ± 17.56			7.55 ± 2.19		
**Lives with grandchildren**	No	80.50 ± 19.08	0.56	0.61	7.41 ± 2.34	−0.37	0.71
	Yes	77.86 ± 17.37			7.64 ± 2.56		
**Lives with other family members**	No	79.98 ± 18.23	−1.09	0.23	7.34 ± 2.31	−1.42	0.16
	Yes	81.96 ± 21.46			7.64 ± 2.43		
**Lives with other people**	No	80.41 ± 19.05	−0.35	0.73	7.40 ± 2.35	−0.65	0.52
	Yes	82.13 ± 18.85			7.80 ± 1.47		
**Working person**	No	81.17 ± 19.96	0.77	0.43	7.43 ± 2.32	0.66	0.51
	Yes	80.02 ± 18.48			7.18 ± 2.69		
**Financial situation**	Good	83.39 ± 19.43	1.46	0.21	7.42 ± 2.48	3.56	0.01
	Rather good	81.35 ± 18.00			7.75 ± 2.11		
	Average	80.18 ± 19.49			7.43 ± 2.27		
	Rather bad	77.06 ± 16.87			6.64 ± 2.66		
	Bad	84.05 ± 22.64			7.59 ± 2.70		
**Current health status**	Very good	80.81 ± 18.12	0.59	0.66	7.52 ± 2.23	1.92	0.11
	Good	81.49 ± 18.45			7.58 ± 2.15		
	Average	79.60 ± 19.14			7.32 ± 2.35		
	Bad	80.70 ± 19.92			7.39 ± 2.62		
	Very bad	75.57 ± 23.53			5.93 ± 3.27		
**Number of diseases**	None	77.75 ± 18.89	1.53	0.19	7.11 ± 2.31	1.09	0.36
	1 disease	80.45 ± 18.87			7.51 ± 2.21		
	2 diseases	82.54 ± 19.69			7.55 ± 2.34		
	3 diseases	81.71 ± 18.24			7.40 ± 2.62		
	More	80.15 ± 19.06			7.62 ± 2.50		
**Type of visit**	POZ	77.52 ± 19.04	−3.78	<0.001	7.20 ± 2.30	−2.23	0.03
	AOS	82.85 ± 18.71			7.59 ± 2.36		

**Table 3:** Analysis of differences in the perception of visits between outpatient specialist care and primary healthcare.

During the Visit, the Doctor	AOS M ± SD	POZ M ± SD	Statistic *t*-test	*p*-Value
found the cause of my symptoms	4.85 ± 1.85	4.68 ± 1.70	−1.35	0.071
presented me a probable further course of treatment	5.38 ± 1.66	4.79 ± 1.76	−4.59	0.000
discussed the possible consequences of the disease	4.87 ± 1.85	4.46 ± 1.86	−2.94	0.002
presented the results of the research conducted	5.51 ± 1.62	4.80 ± 1.84	−5.45	0.000
gave me advice about the medicines I take	5.18 ± 1.79	4.87 ± 1.79	−2.34	0.008
presented treatment recommendations	5.59 ± 1.49	5.06 ± 1.68	−4.48	0.000
talked to me about how I was feeling and how I was coping	4.97 ± 1.87	4.52 ± 1.95	−3.16	0.001
encouraged me	4.70 ± 1.87	4.34 ± 1.81	−2.64	0.004
showed me concern	5.15 ± 1.69	4.69 ± 1.76	−3.57	0.000
talked to me about what was harmful to my health	4.11 ± 2.03	3.75 ± 2.04	−2.37	0.018
advised me what I could do to improve my functioning in everyday life	4.14 ± 2.04	3.87 ± 1.99	−1.76	0.078
encouraged me to introduce favourable changes	4.13 ± 2.01	3.90 ± 2.06	−1.49	0.141
suggested how to maintain contacts with other people	3.87 ± 2.01	3.84 ± 2.00	−0.21	0.835
talked to me about how I can spend my time actively	3.72 ± 2.00	3.91 ± 2.04	1.22	0.216
told me how to maintain life satisfaction	3.73 ± 1.98	3.91 ± 2.04	1.20	0.232
was kind towards me	4.31 ± 2.03	4.04 ± 2.01	−1.75	0.077
treated me seriously	4.36 ± 2.06	4.00 ± 2.09	−2.30	0.022
showed me respect	4.23 ± 1.99	4.09 ± 2.03	−1.29	0.196

## Data Availability

All data are available from the corresponding author.
